# 

**Published:** 1844-12

**Authors:** 


### THE WESTERN JOURNAL OF MEDICINE AND SURGERY:

EDITED BY

DANIEL DRAKE, M. D.

AND

LUNSFORD P. YANDELL, M. D.

PROFESSORS IN THE LOUISVILLE MEDICAL INSTITUTE,

AND

THOMAS W. COLESCOTT, M. D.

NO. LX.—DECEMBER, 1844.

NEW SERIES.

VOL. II.—NO. VI.

LOUISVILLE, KY.

PUBLISHED MONTHLY, BY PRENTICE AND WEISSINGER,

PROPRIETORS AND PRINTERS.

1844.

*This Number contains 4 sheets—Postage under 100 miles 6 cents, over 100 miles 10 cents*

